# Genetics of digestive efficiency in growing pigs fed a conventional or a high‐fibre diet

**DOI:** 10.1111/jbg.12506

**Published:** 2020-09-20

**Authors:** Vanille Déru, Alban Bouquet, Etienne Labussière, Philippe Ganier, Benoît Blanchet, Céline Carillier‐Jacquin, Hélène Gilbert

**Affiliations:** ^1^ GenPhySE Université de Toulouse INRAE ENVT Castanet Tolosan France; ^2^ France Génétique Porc Le Rheu France; ^3^ IFIP‐Institut du Porc Le Rheu France; ^4^ PEGASE, INRAE Institut Agro Saint‐Gilles France; ^5^ UE3P INRAE Saint‐Gilles France

**Keywords:** digestive efficiency, feed efficiency, genetics, high‐fibre diet, pigs

## Abstract

The use of diets with increased dietary fibre content (HF) from alternative feedstuffs is a solution to limit the impact of increased feed costs on pig production. This study aimed at determining the impact of an alternative HF diet on pig digestibility and at estimating genetic parameters of this trait. Digestibility coefficients (DC) of energy, organic matter and nitrogen were predicted from faecal samples analysed with near infrared spectrometry for 1,242 samples, and it represented 654 Large White pigs fed a conventional (CO) diet and 588 fed a HF diet. Growth and feed efficiency traits, carcass composition and meat quality traits were recorded. Pigs fed the HF diet had significantly lower DC than pigs fed the CO diet (−4.5 to 6.0 points). The DC were moderately to highly heritable (about 0.26 ± 0.12 and 0.54 ± 0.15 in the CO and the HF diet, respectively). Genetic correlations were favourable with feed conversion ratio, daily feed intake and residual feed intake, but unfavourable with average daily gain (ADG) and carcass yield (CY). To conclude, DC could be an interesting trait to include in future breeding objectives if pigs were fed diet with HF diets, but adverse genetic trends with ADG and CY would have to be taken into account.

## 1 INTRODUCTION

Incorporating cheaper and more fibrous feedstuffs in pig diets could be a solution to improve the robustness of the pig industry to feed cost volatility and competition between feed, food and fuel industries for crop production. However, pigs fed a diet with increased dietary fibre contents have lower performances for growth, feed efficiency (Levasseur et al., 1998; Quiniou & Noblet, 2012; Sevillano et al., 2018) and carcass yield (CY) (Déru et al., 2020). This reduction in feed efficiency may be explained by the negative impact of increased fibre content on digestibility of nutrients in growing pigs (Le Gall et al., 2009; Le Goff et al., 2002; Mauch et al., 2018), whereas the development of digestive tract decreased CY (Jarrett & Ashworth, 2018). It would thus be desirable to select pigs able to more efficiently digest dietary fibres. If genetic variability for digestive efficiency traits has been found in broilers (Mignon‐Grasteau et al., 2004), only few evidence has been reported in pigs, on limited numbers of animals (Hardie et al., 2014; Noblet et al., 2013). In poultry, heritabilities estimated for digestibility coefficients (DC) were much higher for a diet containing rialto wheat, that is more difficult to digest than a corn‐based diet (Mignon‐Grasteau et al., 2004). So far, the genetic analysis of digestive efficiency in growing pigs has been limited by the ability to measure individual DC on a large number of animals. Indeed, the gold standard method requires isolating pigs in digestibility cages to collect urine and faeces separately. To alleviate extensive and expensive laboratory chemical dosages of nutrients, near infrared spectrophotometry (NIRS) can be used to predict the chemical composition of both feed and faeces in most farm animals, as reviewed by Bastianelli et al. (2018). In some studies, NIRS was used to directly predict DC from a total collection of faeces for pigs raised in individual cages in combination with the use of indigestible markers in the feed (Bastianelli et al., 2015). To facilitate measuring DC in farm conditions, that is without indigestible markers and avoiding total collection of faeces, Labussière et al. (2019) proposed to predict individual DC for energy, organic matter and nitrogen from NIRS spectra based on spot sampling of faeces. With this method, predictions of DC were sufficiently accurate to rank animals according to their digestive efficiency, especially when feed contained a large amount of dietary fibres. This methodology was applied in our study to evaluate the potential of NIRS‐based predictions to select for better digestive efficiency in pigs, as a new tool to improve feed efficiency of pigs exposed to increased levels of dietary fibres.

The main objective of this study was to estimate the genetic parameters of DC for nitrogen, organic matter and energy, as well as their genetic relationships with other production traits in a Large White (LW) pig population. Because we hypothesized that the genetic variability of DC could vary with the feed characteristics, as observed in broilers, two groups of relatives were fed either a conventional (CO) diet or a less digestible diet with increased dietary fibre content (HF diet). Finally, the last objective was to evaluate, from a genetic point of view, whether the current selection on feed efficiency indirectly selects pigs with the best digestive efficiency.

## 2 MATERIALS AND METHODS

### 2.1 Experimental design

The study was conducted in accordance with the French legislation on animal experimentation and ethics. The certificate of Authorization to Experiment on Living Animals was issued by the Ministry of Higher Education, Research and Innovation to conduct this experiment under reference number 2017011010237883 at INRA UE3P—France Génétique Porc phenotyping station (UE3P, INRA, 2018. Unité expérimentale Physiologie et Phénotypage des Porcs, France, https://doi.org/10.15454/1.5573932732039927E12).

#### 2.1.1 Animals

**FIGURE 1:**
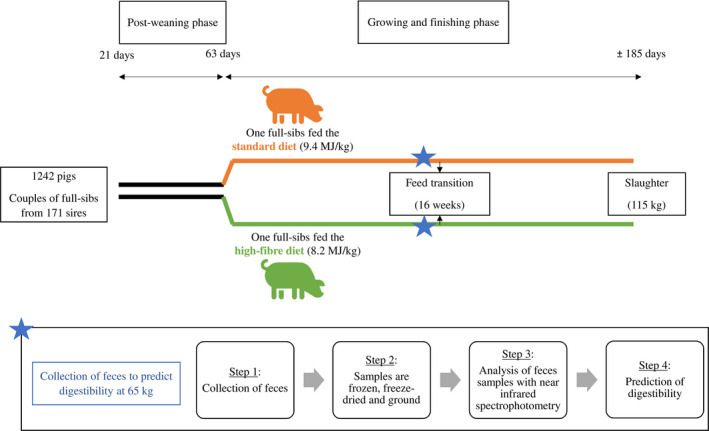
Experimental design [Colour figure can be viewed at wileyonlinelibrary.com]

A total of 1,942 purebred LW male pigs were reared in 35 successive batches in 2017 and 2018 at the INRAE UE3P France Génétique Porc phenotyping station. A family structure was organized by preferentially testing pairs of full‐sibs, each sib being fed one of the diets to facilitate the estimation of genetic covariances across diets in a data set of limited size. Housing conditions and management of pigs were described in Déru et al. (2020). Upon arrival, full‐sibs were separated and allotted in pens of 14 animals. Pigs were raised in postweaning facilities until 9 weeks of age and fed a standard two‐phase postweaning dietary sequence. Then, they were moved to the growing‐finishing facilities without mixing, one of the siblings started to be fed the CO diet and the other one the HF diet, whose compositions are detailed in the next section. Each growing–finishing pen contained a single place electronic feeder equipped with a weighing scale (Genstar, Skiold Acemo) to record individual feed intake and body weight of the animal at each visit to the feeder. At 115 kg body weight, pigs were fasted for 24 hr and then transported to the slaughterhouse. Animals were slaughtered in 89 slaughter batches of around 19 pigs. All pigs were issued from 171 sires representative of those used in the French LW collective breeding scheme, and each couple of full‐sibs came from a different dam. Pigs that experienced health problems or injury during the test period, in equal proportion in the two diets, were discarded from the analysis. At this step, 1,663 pigs were kept in the data set with 880 pigs fed a CO dietary sequence and 783 pigs fed a HF dietary sequence. The design of the experiment is presented in Figure 1.

#### 2.1.2 Diets

During the growing–finishing phase, the two sets of pigs were fed a two‐phase dietary sequence. A growing type of diet was first distributed, and then, a 5‐day transition was organized at 16 weeks of age and a finishing diet was provided until the end of test (slaughter body weight). The CO dietary sequence was formulated to cover energy and amino acids requirements of pigs. The CO and HF diets had ingredient compositions close to those used by Labussière et al. (2019), to ensure maximal accuracy of NIRS digestibility predictions. It included soluble dietary fibres, with sugar beet pulp, and insoluble dietary fibres, with wheat bran and soybean hulls. The detailed composition of CO and HF feeds is described in Table S1. Based on feed formulation, the diets differed in net energy (NE), with 9.6 MJ/kg for the CO diet and 8.2 MJ/kg for the HF diet, and in neutral detergent fibre (NDF), with 13.90% for the CO diet and 23.95% for the HF diet. The standardized ileal digestibility was identical in both dietary sequences, to 0.94 g/MJ NE in the growing phase and to 0.81 g/MJ NE in the finishing phase.

#### 2.1.3 Traits

For each animal, growth and feed efficiency traits were computed between 35 and 115 kg, namely the average daily gain (ADG), the daily feed intake (DFI) and the feed conversion ratio (FCR). Carcass composition was measured 24 hr after slaughter. They comprised lean meat percentage (LMP), calculated from primal cut weights using the equation defined by Daumas (2008), and CY. Primal cut weights were expressed relative to the carcass weight as the belly percentage (bellyP), the loin percentage (loinP), the shoulder percentage (shoulderP), the backfat percentage (backfatP) and the ham percentage (hamP). Furthermore, meat quality traits were also recorded 24 hr after slaughter. As described in Déru et al. (2020), they comprised the ultimate pH (upH) measured on the *semi‐membranous* muscle of the ham, as well as the lightness (L*), the redness (a*) and the yellowness (b*) of the meat measured on the *gluteus superficialis* muscle using a Minolta Chromameter CR300.

Residual feed intake (RFI) was determined using a single multiple linear regression (R Core Team, 2016) for the two diets, of DFI on ADG, LMP, CY and average metabolic body weight as described in Déru et al. (2020).

### 2.2 Prediction of digestibility coefficients with the NIRS method

#### 2.2.1 Sample collection and preparation

To determine DC, faecal samples (about 50 g) were collected individually at 16 weeks of age, just before the feed transition between the growing and finishing phases. They were manually homogenized and then stored at −20°C in plastic containers until further analysis. Then, samples were freeze‐dried and ground with a grinder (Grindomix GM200, Retsch). Ground samples were stored at +4°C before analysis with NIRS. In total, 1,412 pigs of our experiment had valid performances and DC.

#### 2.2.2 NIRS predictions of digestibility coefficients

Digestibility coefficients of energy, organic matter and nitrogen were predicted based on the prediction protocol described by Labussière et al. (2019). The prediction equations of the three DC were previously obtained using a calibration data set of 412 samples for DC of organic matter, 424 samples for DC of nitrogen and 423 samples for DC of energy, with *R* ^2^ higher than .89, very low biases (−0.01 to 0.00) and regression coefficients between predicted and true values close to one. The prediction equations were assessed by comparison with values measured using the total collection technique in individual digestibility cage on 10 pigs that were fed the two diets. Then, three successive spectra were acquired for each sample, and the average spectrum was used for prediction of DC. Usual criteria were used to individually assess the quality of NIRS predictions and detect outliers. When prediction values were lower or higher than the bounds of the model, samples were eliminated (131 samples). During the partial least squares regression, a Mahalanobis distance was calculated for each prediction with the OPUS/Quant2 software (Bruker) previously used to determine DC. The 99.999% distribution bound of the calibration data set was used as a reference and multiplied by three to obtain a realistic prediction interval for the variability of an independent data set. Thus, if the Mahalanobis distance of a prediction was greater than the realistic prediction interval, samples were removed from the data set (39 samples). It represented a bound of 0.36 for DC of organic matter, 0.24 for DC of nitrogen and 0.33 for DC of energy. After data cleaning, 1,242 samples were obtained for 654 pigs fed the CO diet and 588 pigs fed the HF diet having also all other growth and feed efficiency measurements.

### 2.3 Statistical analyses

#### 2.3.1 Phenotypic comparison of digestibility coefficients across diets

First, a statistical analysis was carried out to assess the differences of DC between diets. The model contained the fixed effects of the batch and diet, as well as the random effect of the pen nested within the diet and batch and the random effect of the sire. Because feed intake influences the DC of dry matter and crude protein (Verschuren, et al., 2019), the individual DFI was also included in the models as a covariate nested within diet. A Levene test was used to evaluate the heterogeneity of residual variances among diets for each DC using the ANOVA procedure ((SAS, 2013) version 9.4; SAS Institute Inc.). In case of variance heteroscedasticity among diets, residual variances were estimated within each diet. Least squares means (LSMeans) of DC were determined for each diet using the MIXED procedure ((SAS, 2013) version 9.4; SAS Institute Inc.). The significance of differences between diet LSMeans was evaluated using a Student *t* test with the SAS software ((SAS, 2013) version 9.4; SAS Institute Inc.).

#### 2.3.2 Genetic analyses

A genetic analysis was undertaken to estimate the genetic variance of DC as well as their genetic correlations with other production traits. In the first instance, all traits were analysed separately within diet with the following linear mixed model:

$$y=Xß+\mathrm{Zu}+\left(\mathrm{Wn}\right)+\left(\mathrm{Sq}\right)+e,$$

where **y** is the vector of phenotypes for a given trait, and **ß** is the vector of fixed effects depending on the trait considered: batch and DFI for DC, batch and weight at the end of postweaning phase for ADG and FCR, batch and weight at the end of test for DFI, batch and half carcass weight for LMP and primal cut proportions, and batch and hot carcass weight with head for CY and meat quality traits. **X** is the incidence matrix relating observations to fixed effects. **Z** is the incidence matrix of the additive genetic effects. **u** ~ *N*(0, **A** $\sigma_{u}^{2}$) is the vector of additive genetic effects for the considered trait, where **A** is the pedigree relationship matrix built tracing back five generations of pedigree and $\sigma_{u}^{2}$ is the additive genetic variance. **n** ~ *N*(0, **I** $\sigma_{n}^{2}$) is the random effect of the pen effect nested within diet and batch, applied to all traits except primal cut ratios. **W** is an incidence matrix relating performances to the random effect **n**. **q** ~ *N*(0, **I** $\sigma_{q}^{2}$) is the random effect of the slaughter dates applied only for upH. **S** is an incidence matrix relating performances to slaughter date effect. Finally, **e** ~ *N*(0, **I** $\sigma_{e}^{2}$) is the residual random effect. Variance components were estimated by average information restricted maximum likelihood (AIREML) using the ASREML 3.0 software (Gilmour et al., 2009). Then, bivariate analyses were carried out to estimate covariances between DC and other production traits within diets, and between diets for DC. In this study, heritability estimates were qualified as low below 0.20, moderate from 0.20 to 0.40 and high above 0.40. Genetic correlations were considered low for absolute values between 0.00 and 0.20, moderate between 0.20 and 0.50 and high above 0.50.

In a second instance, as genetic correlations estimated across diets for production traits were high (Déru et al., 2020), genetic correlations between DC and other production traits were estimated by pooling performances from pigs fed the CO and the HF diet, including the diet as an additional fixed effect in the linear mixed models previously defined.

Then, because DFI may not be always available in practical applications, genetic variances were also estimated for DC traits without adjusting for DFI, as well as their genetic covariances with other production traits in each diet and for the two diets combined.

Finally, to evaluate whether digestive efficiency was improved with selection on FCR, we compared the ranks of sires based on standardized estimated breeding values (SEBV) obtained from univariate analyses of FCR and the three DC. Only 77 sires with reliability of estimated breeding values higher than 0.40 for all traits were kept for analyses. Ranks were compared using Spearman rank correlations. The 95% confidence intervals (CI) were determined for each rank correlation using a bootstrap approach implemented in the spearman.ci function on R (R Core Team, 2016), with 1,000 replicates. Finally, we applied the same procedure to evaluate whether the adjustment of DC for DFI had an impact on the rank correlations of DC.

## 3 RESULTS

### 3.1 Effects affecting digestibility coefficients

**FIGURE 2:**
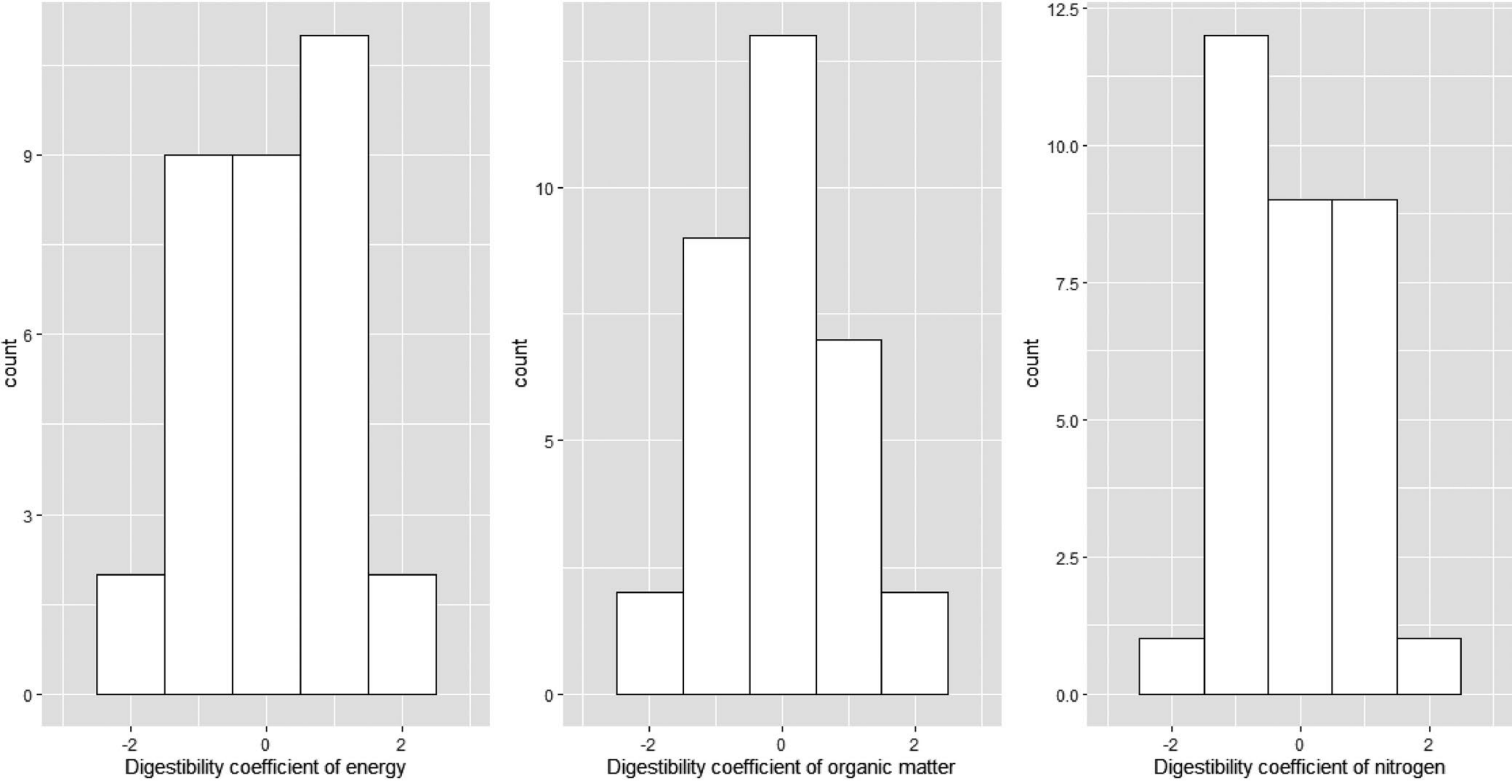
Distribution of the estimates of the batch effect for the three digestibility coefficients (*N* = 33)

Residual variances were homogenous between diets for DC of energy (*p* = .14) and organic matter (*p* = .50) and heterogeneous for DC of nitrogen (*p* = .03). The batch (Figure 2), the diet and DFI within diet had significant effects on DC (*p* < .0001; Table 1). The LSMeans of energy, nitrogen and organic matter DC were higher in the CO diet (82.0% on average) than in the HF diet (76.7% on average), with a contrast of +6, 4.6 and 5.5 points, respectively (*p* < .001; Table 1). In addition, they were lower for nitrogen than for energy and organic matter.

**TABLE 1:** Least square means (LSMeans) of digestibility coefficients for growing pigs fed the conventional (CO) or the high‐fibre (HF) diet and estimates of the regression coefficient for daily feed intake nested within diet, along with their standard errors (*SE*) and percentage of variance of the pen within batch within diet and sire effects^1^

	Diet		DFI × diet (%/kg feed)		Pen × batch × diet		Sire	
	LSMeans (*SE*)		Estimate (*SE*)		% variance		% variance	
	CO diet	HF diet	CO diet	HF diet	CO diet	HF diet	CO diet	HF diet
Energy, %	84.2^a^ (0.15)	78.2^b^ (0.16)	−2.65^a^ (0.40)	−2.01^b^ (0.38)	15^2^		6^3^	
Nitrogen, %	78.5^a^ (0.16)	73.9^b^ (0.16)	−4.49^a^ (0.48)	−2.82^b^ (0.41)	8	10	5	6
Organic matter, %	83.4^a^ (0.14)	77.9^b^ (0.14)	−2.42^a^ (0.37)	−1.82^b^ (0.35)	13^2^		6^3^	

^a,b^ Means in the same row with different superscript are statistically different according to Student test (*p* < .05).

^1^ From a linear mixed model including the fixed effects of the batch, the diet, the covariate of daily feed intake within diet and the random effect pen within batch within diet. All effects were significant for *p* < .0001.

^2^ The residual variances were homogenous between diets for digestibility coefficients of energy and organic matter; thus, the percentage of variance explained by the effect pen within batch within diet was identical between diets for these traits.

^3^ The residual variances were homogenous between diets for digestibility coefficients of energy and organic matter; thus, the percentage of variance explained by the sire effect was identical between diets for these traits.

The random effect of pen within batch within diet explained from 8% to 15% of the trait variances and the random effect of sire explained from 5% to 6% of the trait variances, depending on the DC (*p* < .0001). The estimates of the batch effects (Figure 2) covered more than two phenotypic standard deviations (Table 2) for each DC. Finally, the regression coefficients of DFI within diet were significantly higher in the CO diet, ranging from −4.49 to −2.42% DC/kg feed in the CO diet and from −2.82 to −1.82% DC/kg feed in the HF diet. Given these preliminary results, variance components were estimated while adjusting the DC with DFI in all analyses, unless stated otherwise.

**TABLE 2:** Heritability (*h* ^2^), genetic and phenotypic variances of digestibility coefficients adjusted for daily feed intake, for growing pigs fed the conventional (CO) and high‐fibre (HF) diet, along with their standard error (*SE*)

	CO diet			HF diet			Across diets
	*h* ^2^ (*SE*)	genetic variance (*SE*)	Phenotypic variance (*SE*)	*h* ^2^ (*SE*)	Genetic variance (*SE*)	Phenotypic variance (*SE*)	Genetic correlation (*SE*)
Digestibility coefficients							
Energy, %	0.26 (0.12)	1.19 (0.55)	4.58 (0.35)	0.54 (0.15)	2.83 (0.87)	5.28 (0.37)	0.71 (0.20)
Nitrogen, %	0.27 (0.12)	1.96 (0.86)	7.03 (0.50)	0.56 (0.15)	3.29 (1.00)	5.93 (0.42)	0.85 (0.21)
Organic matter, %	0.27 (0.12)	1.06 (0.47)	3.85 (0.30)	0.54 (0.15)	2.41 (0.75)	4.49 (0.32)	0.76 (0.20)

### 3.2 Variance components for digestibility coefficients

Heritability, genetic and phenotypic variances of the three DC are presented in Table 2. Estimated heritabilities of the three DC were moderate in the CO diet (about 0.27 ± 0.12) to high in the HF diet (about 0.55 ± 0.15), with similar estimates for the three DC within diets. Phenotypic variances of DC of energy and organic matter were larger in the HF than in the CO diet. On the contrary, the phenotypic variance was larger in the CO diet for nitrogen DC. Estimated genetic variances were always higher in the HF diet than in the CO diet. Therefore, DC heritabilities were higher in the HF diet for the three DC.

For all DC, genetic correlations between the HF and CO diets were high, ranging from 0.71 ± 0.20 to 0.85 ± 0.21, and not different from one or 0.80, taken as a reference value to consider traits as genetically different between diets, given the standard errors (Table 2). The phenotypic correlations between DC with the HF and CO diets were higher than 0.89. Genetic correlations between the three DC within diet were very high, ranging from 0.96 to 0.99.

Genetic correlation estimates of DC with production traits in each diet, and for both diets combined, are presented in Table 3. Results reported in the text are given only for estimates obtained in each diet. Genetic correlations between the three DC and FCR were negative and favourable in the CO and the HF diets, from −0.56 ± 0.28 to −0.16 ± 0.39, and also favourable with RFI (from −0.99 ± no estimate [NE] to −0.50 ± 0.25). Genetic correlations between the three DC and DFI were high for pigs fed the CO diet (from −0.73 ± 0.20 to −0.59 ± 0.21) and moderate to high for pigs fed the HF diet (from −0.58 ± 0.28 to −0.41 ± 0.30). Genetic correlations between DC and ADG were negative and unfavourable and varied from −0.33 ± 0.31 to −0.30 ± 0.30 and from −0.57 ± 0.37 to −0.30 ± 0.36 in the CO and the HF diet, respectively.

**TABLE 3:** Genetic correlations between digestibility coefficients (DC) adjusted for daily feed intake and production traits for growing pigs fed a conventional (CO) diet or a high‐fibre (HF) diet and for the two data sets combined, along with their standard errors (*SE*)

	Energy DC		Nitrogen DC		Organic Matter DC		Diets combined		
	CO diet	HF diet	CO diet	HF diet	CO diet	HF diet	Energy	Nitrogen	Organic Matter
Growth traits									
FCR, kg/kg	−0.56 (0.28)	−0.16 (0.39)	−0.27 (0.27)	−0.34 (0.38)	−0.53 (0.28)	−0.21 (0.39)	−0.27 (0.17)	−0.24 (0.10)	−0.23 (0.17)
DFI, kg/day	−0.73 (0.20)	−0.57 (0.28)	−0.59 (0.21)	−0.41 (0.30)	−0.72 (0.19)	−0.58 (0.28)	−0.51 (0.17)	−0.53 (0.13)	−0.45 (0.17)
ADG, g/day	−0.30 (0.30)	−0.57 (0.37)	−0.33 (0.31)	−0.30 (0.36)	−0.33 (0.29)	−0.53 (0.37)	−0.34 (0.17)	−0.15 (0.17)	−0.34 (0.17)
RFI, g/day	−0.99^a^	−0.62 (0.23)	−0.83 (0.40)	−0.50 (0.25)	−0.99^a^	−0.62 (0.23)	−0.66 (0.16)	−0.54 (0.16)	−0.56 (0.16)
Carcass Composition									
LMP, %	0.09 (0.30)	−0.14 (0.24)	−0.05 (0.29)	−0.22 (0.23)	0.13 (0.29)	−0.16 (0.24)	−0.02 (0.17)	−0.08 (0.16)	0.02 (0.16)
Carcass Yield, %	−0.32 (0.29)	−0.15 (0.23)	−0.28 (0.29)	−0.27 (0.22)	−0.29 (0.29)	−0.20 (0.22)	−0.32 (0.19)	−0.49 (0.33)	−0.31 (0.17)
BellyP, %	0.34 (0.35)	0.28 (0.34)	0.36 (0.33)	0.12 (0.34)	0.24 (0.34)	0.22 (0.35)	0.23 (0.19)	0.20 (0.18)	0.22 (0.19)
LoinP, %	−0.16 (0.42)	−0.31 (0.34)	−0.41 (0.39)	−0.15 (0.35)	−0.14 (0.41)	0.22 (0.35)	−0.10 (0.23)	0.01 (0.20)	−0.10 (0.23)
ShoulderP, %	−0.40 (0.32)	−0.09 (0.27)	−0.18 (0.31)	−0.05 (0.25)	−0.37 (0.31)	−0.09 (0.27)	−0.19 (0.17)	−0.16 (0.17)	−0.18 (0.17)
BackfatP, %	−0.10 (0.29)	0.09 (0.25)	−0.04 (0.29)	−0.09 (0.24)	−0.14 (0.29)	0.04 (0.25)	−0.06 (0.16)	0.02 (0.15)	−0.05 (0.16)
HamP, %	0.30 (0.31)	0.06 (0.30)	0.18 (0.28)	−0.17 (0.29)	0.40 (0.29)	0.02 (0.30)	0.09 (0.18)	−0.02 (0.17)	0.10 (0.18)
Meat quality									
upH	−0.82 (0.37)	−0.31 (0.40)	−0.41 (0.38)	−0.55 (0.41)	−0.78 (0.37)	−0.32 (0.40)	−0.26 (0.17)	−0.03 (0.22)	−0.23 (0.22)
L*	0.09 (0.38)	−0.11 (0.41)	−0.14 (0.35)	0.49 (0.51)	0.11 (0.37)	−0.06 (0.41)	−0.07 (0.23)	−0.03 (0.24)	−0.08 (0.23)
a*	0.34 (0.26)	−0.16 (0.22)	0.16 (0.26)	−0.20 (0.21)	0.29 (0.25)	−0.21 (0.22)	0.11 (0.08)	−0.18 (0.15)	0.07 (0.08)
b*	0.28 (0.39)	−0.16 (0.33)	0.06 (0.37)	0.09 (0.35)	0.25 (0.38)	−0.20 (0.33)	−0.12 (0.19)	−0.16 (0.20)	−0.16 (0.19)

Abbreviations: a*, redness of the meat; ADG, average daily gain; b*, yellowness of the meat; BackfatP, backfat percentage; BellyP, belly percentage; DFI, DAILY FEED INtake; FCR, feed conversion ratio; HamP, ham percentage; L, lightness of the meat; LMP, lean meat percentage; LoinP, loin percentage; RFI, residual feed intake; ShoulderP, shoulder percentage; upH, ultimate pH 24 hr after the slaughterhouse.

^a^ Estimated correlation and at the edge of the parameter space

Genetic correlations between DC and LMP were not different from zero in the CO diet (from −0.05 ± 0.29 to 0.13 ± 0.29) and also close to zero in the HF diet (from −0.22 ± 0.23 to −0.14 ± 0.24). Genetic correlations were close to zero with other carcass composition traits in both diets. In addition, genetic correlations between DC and meat quality traits were close to zero for all traits except upH, that had negative genetic correlations with DC, ranging from −0.82 ± 0.37 to −0.41 ± 0.38 and from −0.55 ± 0.41 to −0.31 ± 0.40 in the CO and the HF diet, respectively.

In general, the magnitude of the correlations did not significantly differ between the diets. When the performances recorded with the CO and HF diets were analysed together, consistent results were obtained for most traits, with reduced standard errors. However, correlations with CY tended to differ from zero with these new estimates, and the correlations with upH were reduced to less extreme values.

### 3.3 Impact of daily feed intake on the genetic variability of digestibility coefficients

Analysing DC without adjusting for DFI in the linear mixed models had a substantial impact on the DC estimated variance components in the CO diet (Table S1). Indeed, both genetic and phenotypic variances estimated with this model increased in the CO diet, resulting in larger heritability estimates for DC than with DFI adjustment. On the contrary, in the HF diet, the estimated genetic parameters were very close to those of DC adjusted for DFI. Finally, the genetic correlations estimated between DC across diets were still high, ranging from 0.71 ± 0.20 to 0.85 ± 0.21.

Analysing DC without adjusting for DFI also had a slight impact on the genetic correlations estimated with production traits (Table S2). In analyses with both diets combined, genetic correlations were close to those estimated for DC adjusted for DFI, except for three traits: genetic correlations were negative and of higher magnitude with DFI (from −0.75 ± 0.10 to −0.64 ± 0.10), with ADG (from −0.53 ± 0.13 to −0.42 ± 0.13) and with upH (from −0.42 ± 0.23 to −0.21 ± 0.21).

### 3.4 Rank correlations

The rank correlations between sires SEBV of FCR and the three DC adjusted for DFI are presented in Table 4 along with their 95% CI. They were moderate, ranging from −0.25 to −0.21, and their 95% CI varied between −0.47 and 0.03. Moreover, the rank correlations estimated between sires SEBV of the three DC without adjustment for DFI were higher than 0.95 with 95% CI spanning from 0.90 to 0.98 (Table 4).

**TABLE 4:** Spearman rank correlations of estimated breeding values between the digestibility coefficients (DC) and between digestibility coefficients and the feed conversion ratio (FCR)

	Spearman correlation	95% Confidence interval	
		Lower	Upper
SEBV_DC_E_DFI – SEBV_FCR	−0.25	−0.47	0.03
SEBV_DC_N_DFI – SEBV_FCR	−0.21	−0.43	0.03
SEBV_DC_OM_DFI – SEBV_FCR	−0.25	−0.46	0.02
SEBV_DC_E – SEBV_DC_E_DFI	0.97	0.91	0.98
SEBV_DC_N – SEBV_DC_N_DFI	0.97	0.93	0.98
SEBV_DC_OM – SEBV_DC_OM_DFI	0.95	0.90	0.98

Abbreviations: SEBV_DC_E, estimated breeding values of digestibility coefficient of energy not adjusted for DFI, standardized by their genetic standard deviation; SEBV_DC_E_DFI, estimated breeding values of digestibility coefficient of energy adjusted for DFI, standardized by their genetic standard deviation; SEBV_DC_N, estimated breeding values of digestibility coefficient of nitrogen content not adjusted for DFI, standardized by their genetic standard deviation; SEBV_DC_N_DFI, estimated breeding values of digestibility coefficient of nitrogen content adjusted for DFI, standardized by their genetic standard deviation; SEBV_DC_OM, estimated breeding values of digestibility coefficient of organic matter not adjusted for DFI, standardized by their genetic standard deviation; SEBV_DC_OM_DFI, estimated breeding values of digestibility coefficient of organic matter adjusted for DFI, standardized by their genetic standard deviation; SEBV_DFI, estimated breeding values of daily feed intake, standardized by their genetic standard deviation; SEBV_FCR, estimated breeding values of feed conversion ratio, standardized by their genetic standard deviation.

## 4 DISCUSSION

Applying a novel methodology to predict DC from NIRS analyses of faecal samples, the findings presented in this study confirmed that digestive efficiency of nitrogen, energy and organic matter are heritable traits in pigs and that they are genetically correlated to other feed efficiency traits. Furthermore, the genetic variability seemed influenced by the type of feed used to rear pigs, with larger heritability estimated when the feed had larger fibre contents.

### 4.1 Advantages and limits of using NIRS analysis of faeces to predict digestibility

Analysing faecal spot samples with NIRS seems to be a promising methodology to predict DC on a large number of animals for breeding applications. Accurate NIRS predictions of faecal nutrient and energy DC were previously reported in pigs (Bastianelli et al., 2015), but they were based on total collection of faeces, which cannot be envisaged in farm conditions. To alleviate this limitation, Labussière et al. (2019) showed that DC could be predicted with NIRS from faeces obtained by spot sampling with a reasonable loss of accuracy (validation *R* ^2^ > 85%). Moreover, this method presents several advantages compared to the gold standard: (a) it is not necessary to isolate pigs in digestibility cages which is beneficial for welfare aspects but also because the housing system (individual vs. group housing) influences digestibility measurement (De Haer & de Vries, 1993), and (b) the cost of the measure is strongly reduced because chemical analyses are not required anymore.

However, predicting digestibility from faecal samples obtained from spot collection assumes that samples are representative enough of a total collection. Diurnal variations of faeces composition may influence digestibility measurements (Horvath et al., 1958; Moore, 1958). Using a single faecal sample of 24 g, Moore (1958) showed that errors were moderate for estimating daily DC of crude protein and dry matter using indigestible markers. Measurement errors were even reduced when the quantity of sampled faeces was larger. For other components of the feed (crude fibre, ash), the predictions of DC were shown to be inaccurate when based on spot sampling (Moore, 1958; INRAE, 2019). As a result, predictions of DC were not considered for crude fibre in this study.

Other factors may influence the precision of NIRS‐based predictions of DC, in particular the age at sampling and feed characteristics. Digestive efficiency varies depending on the age of the animal (Noblet et al., 2013; Ouweltjes et al., 2018). INRAE (2019) stated that NIRS‐based DC predictions were adequate for pigs heavier than 60 kg in case of spot sampling. They also showed that, without indigestible markers, the accuracy of the DC predictions was higher when pigs were fed a diet with a high‐fibre content, that is a diet that induced variability in digestive utilization. These authors also insisted on the importance of having connectedness between the calibration and predicted data set to ensure predictions of sufficient accuracy. For our study, the equations used to predict the DC of organic matter, energy and nitrogen had high *R* ^2^, >.89.

In our study, different measures were taken to isolate the “animal factor of variation of digestibility,” as termed by Bastianelli et al. (2015), and to limit the impact of other factors of variation on estimations of genetic parameters. All samples were collected at fixed time in the morning to get a large quantity of faeces (>50 g). Faecal samples were homogenized before storage, and three successive spectra were acquired on freeze‐dried samples and the averaged spectrum provided the predictions for each sample. In addition, the calibration set was completed with faecal samples from pigs related to those used in our experiment and fed the same feeds. The difference between the average DC predicted for the CO and HF diets was consistent with other studies (Le Gall et al., 2009; Le Goff et al., 2002; Mauch et al., 2018). Indeed, Le Goff and Noblet (2001) observed that the DC of energy decreased about 1% per additional NDF point. In our experiment, the DC of energy decreased by 0.6% per point of additional NDF but this lower value may be due to the fact that pigs were fed ad libitum whereas feeding is generally restricted in digestibility experiments. Further research is needed to assess the sensitivity of genetic parameters estimations to the different factors and provide guidelines for use of the method in selection farms.

### 4.2 Genetic and phenotypic variability of nitrogen, energy and organic matter digestibility

Based on a large data set representative of a commercial pig population, heritabilities estimated in both diets confirm that digestive efficiency is a heritable trait, as suggested by Noblet et al. (2013). Estimation of genetic variability suggests that the NIRS predictions make it possible to capture relevant genetic information from faecal samples. Moderate heritabilities were estimated for DC in the CO diet, and larger estimates were obtained in the HF diet. The range of values obtained for the three DC is of similar magnitude as estimations for other production traits. Heritabilities estimated for the DC of dry matter and energy were slightly larger than those estimated by Hardie et al. (2014) which is, to our knowledge, the only study reporting heritability for DC in pigs. This study was based on 122 young pigs infected by the porcine reproductive and respiratory syndrome virus and fed a corn–soybean meal diet with low dietary fibre content (*h* ^2^ = 0.17 ± 0.22 and 0.15 ± 0.23 for dry matter and energy, respectively). However, due to large standard errors Hardie et al. (2014) concluded that the traits were not heritable. In broilers, heritabilities for DC of proteins, starch and lipids of 0.29 ± 0.02, 0.28 ± 0.02 and 0.25 ± 0.02, respectively, were estimated for individuals fed a rialto wheat known for its low digestibility. In this same trial, heritabilities were much lower (0.09 ± 0.02, 0.26 ± 0.05 and, 0.04 ± 0.01) when birds were fed a European conventional corn and soya bean diet, much easier to digest (Mignon‐Grasteau et al., 2010).

As for broilers, genetic parameters were influenced by the diet although significant heritabilities were estimated for the CO diet. The conventional diet used in our study seemed to contain enough dietary fibres (NDF content of 13%) to generate significant genetic variability for DC. The higher heritabilities for DC of energy and organic matter under the HF diet were related to higher genetic variances. On the other hand, genetic variances for DC of nitrogen were close between diets but the phenotypic variance was larger in the CO diet. Pig populations have been selected on a CO diet for many years now. Increasing the fibre content in the feed could create a dietary challenge that exacerbated the variability of the ability of pigs to cope with the feed, resulting in increased genetic variances. This HF diet was formulated to be as generic as possible, including various types of fibres (solubles and insolubles), and, thus, can represent a variety of diets that can be encountered when diversifying ingredient resources. Finally, the accuracy of DC predictions was expected to be larger in the HF diet than in the CO diet (INRAE, 2019).

Feed and genetics explained some of the variability of digestive efficiency, but other important factors could be identified. The batch effect was significant, but certainly limited in our trial by the standardization of the feed resources. In addition, the effect of the pen, nested within the batch and diet, was significant. This pen effect may capture the effect of birth herd comprising a common initial environment and shared intestinal microbiota acquired during the first days of life, that could have an impact on the later digestive efficiency of the pigs, as the gut microbiota appears to play an important role in the digestibility of nutrients in pigs (Niu et al., 2015). In addition, the pen effect would also capture group differences resulting from social interactions and dynamics within groups.

Finally, DFI had a significant effect on predicted DC in both diets. According to our estimations, pigs with larger DFI had lower digestibility of nitrogen and energy. This relationship between feed intake and digestibility of energy and nutrients had already been reported in pigs (Verschuren, et al., 2019). An hypothesis could be that increased DFI is related to faster rates of passage, which in turn reduces absorption in the intestine due to reduced exposure to microbial activity (Cunningham et al., 1962). When analysing DC traits without correcting for DFI, the genetic variance of DC was inflated in the CO diet, which suggested that part of the genetic variability of digestive efficiency was explained by feed intake differences between individuals. In the HF diet, the genetic variance remained constant. The relationship between digestive efficiency and feed intake can be controlled by adding DFI as a covariate in the model when it is available. If not available, different sets of genetic parameters would have to be considered, in particular with a conventional type of diet.

### 4.3 Genetic correlations with production traits

In this study, moderate to high genetic correlations were observed with DFI and RFI whatever the diet. This finding was consistent with the change in digestive efficiency estimated in pigs by Harris et al. (2012) following a selection experiment on RFI, although other selection experiments on this trait did not report an improvement of digestive efficiency in pigs for pigs fed and selected for a non‐fibrous diet (Barea et al., 2010) and in rabbits (Gidenne et al., 2017). These differences could result from the type of feed used for testing and age of the animals and would deserve better understanding.

In our experiment, genetic correlations were low to moderate with FCR. In broilers, negative yet stronger genetic correlations between DC and FCR were reported (from −0.89 to −0.55 for DC of lipids, starch and proteins) (Mignon‐Grasteau et al., 2004). When feed efficiency is quantified by FCR, it captures the efficiency of all biological mechanisms developed by the animal (gross feed efficiency (Knap, 2009)). The RFI is a measure of net feed efficiency, as it is adjusted for the production and maintenance requirements of the pig: it captures all other functions plus individual efficiency deviations for production and maintenance and measurement errors (Kennedy et al., 1993). On the other hand, feed efficiency of growing animals is related to its feed intake, digestive efficiency and metabolic efficiency in the use of the absorbed energy and nutrients for body weight gain (Carré et al., 2008). Thus, digestibility predictions via NIRS offer a direct access to a component of feed efficiency that is never specifically targeted with existing measures, but plays an essential role in the general efficiency of the animal and is usually difficult to measure on large numbers.

An unfavourable genetic correlation was estimated between DC and ADG at the genetic level. This result differed from Hardie et al. (2014), who reported a positive genetic correlation between weight gain and digestibility measurements but estimated with low accuracy. However, as DC had negative genetic correlations with DFI, this correlation could be the indirect effect of reduced feed intake. Moreover, higher digestive efficiency is expected to be related to increased size of the digestive tract, and maintenance requirements for the viscera were estimated to cost three times more energy than muscle (Noblet, Karege, Dubois, & Van Milgen, 1999). Consequently, less energy would be available for growth in pigs with larger gastro intestinal tract, leading to reduced ADG. Similarly, in broilers, a low negative correlation was reported between apparent metabolizable energy and weight gain (Mignon‐Grasteau et al., 2004). Finally, genetic correlations with carcass composition and meat quality were not different from zero when diets were combined, except with CY. The unfavourable genetic correlation between CY and DC may be explained by the larger digestive tract of pigs presenting the best digestive efficiency, but this hypothesis could not be verified in this experiment because the weight of the digestive tract was not available. Thus, the inclusion of digestive efficiency in breeding schemes would have limited impact on carcass composition and meat quality traits, but on CY, that genetic correlations with DC should be accounted for in selection schemes.

Genetic correlations were high between DC within diets, and among diets, so the underlying genetic determinism would be very similar between DC. It could be explained because DC are very related, energy contains organic matter and organic matter contains nitrogen. In addition, selection based on a diet with moderate dietary fibre content would create genetic progress on these traits that would be transferred to pigs fed diets with higher dietary fibre contents. However, selection response would be larger on digestive efficiency if pigs were fed diets with increased contents of dietary fibres.

Finally, all genetic correlations tended to be higher without adjustment for DFI, in particular with feed efficiency, growth traits and upH. So, computation of selection indexes when adjustment for DFI is not possible would require the use of dedicated sets of genetic parameters to properly control the correlated responses on associated traits.

### 4.4 Biological mechanisms explaining variability in digestive efficiency

The high phenotypic correlations between the three DC, especially between DC of organic matter and energy, confirm the high biological relationship between the three DC mentioned before. This result was also found with other methodologies (Hardie et al., 2014) (0.85 ± 0.03).

Some biological mechanisms could be suggested to explain the variability in digestive efficiency. First, feed intake can have a mechanical impact on the digestibility. As indicated above, an increased feed intake can accelerate the rate of passage of digesta and correlatively reduce the digestibility of nutrients and energy. Then, the size of the digestive tract can have an impact on digestive efficiency, with longer digestive tract favouring higher digestibility, as suggested by the negative correlations with CY.

In addition, changes in digestibility could also be related to changes in digestive enzyme activities (Pérez de Nanclares et al., 2017). In this previous study, they observed a lower trypsin activity, an enzyme that participates to protein digestion, in the jejunum for 20 Landrace pigs fed with a high‐fibre rapeseed co‐products diet. In vitro, pure lignin and cellulose have been shown to strongly inhibit pancreatic amylase and trypsin activities (Hansen, 1986). The presence of some types of fibres may reduce enzyme activity and thus explain the fact that nutrients are less digestible for pigs with higher contents of dietary fibres. Finally, the gut microbiota composition variability can also play a role in the variability of digestive efficiency. According to Verschuren, et al. (2019), faecal microbiota can provide a good insight of the role of gut microbiota in total tract nutrient digestion (dry matter, organic matter, crude protein, crude fibre and non‐starch polysaccharides), especially when pigs are fed with fibrous diets (equivalent to our conventional diet). These authors showed that digestive efficiency can be partially predicted by the gut microbiota. All those potential mechanisms will need further exploration to understand the biological bases of digestive efficiency variability.

### 4.5 Practical applications in breeding schemes

Before measuring DC in routine on farms, further work is needed to facilitate sample collections, reduce the associated labour costs and the sampling and laboratory constraints and thus propose procedures applicable to large numbers. For instance, with the current procedure three to four people are required for the faeces collection, which is heavy compared to routine farm tasks, for handling animals and collecting faeces, packaging, freezing at −20°C and managing traceability. For a given batch, the work had to be organized in one single say, preferably early in the morning, to have sufficient quantities of faeces. A strict logistics part (traceability and storage of samples) was necessary to guaranty the samples/ID traceability. An ideal solution would be to analyse the faeces samples directly in the farm using a portable device, as a diagnosis blood analysis, for instance.

In the future, pigs could be fed diets with increasing contents of dietary fibres of several types. The different ranking of animals selected on general feed efficiency criteria (FCR) compared to that of animals selected on digestive efficiency indicates that it would then make a difference to include digestive efficiency in selection objectives to select more efficient animals at the digestive level. With diversifying production systems, having access to the animal capacity to digest could be even more important, but the choice of the best criterion to retain will be essential. In our study, the three DC were highly correlated from a phenotypic and genetic point of view. Pig production contributes to environmental pollution in particular through the excretion and emission of nitrogenous compounds, which could favour a preference for DC of nitrogen to select pigs with improved nitrogen digestive efficiency and thus reduce the impact of pig farming on the environment (Kasper et al., 2020). However, it is not the nitrogen contained in the faeces that contributes most to the environmental pollution, but the nitrogen contained in urine, which is more susceptible to be lost through leaching or runoff (Bindelle et al., 2008).

Even if the capacity for testing feed efficiency in selection schemes can be increased by having more automatic feeders, the costs and associated infrastructure developments remain very significant. Given the high genetic correlations between DFI and DC, digestibility predictions could also be envisaged as an auxiliary trait for feed intake to be available on farm on large numbers of animals without the need of automatic feeders, including production farms if the procedure can be simplified enough in the future. Of course, the relative cost of sampling and measures would have to be compared with those of feeders and put together with their relative accuracies to evaluate the possible genetic gains with different strategies. In addition, the weights applied to the different traits in the selection objective will have to account for unfavourable genetic correlations with DC, and ADG and CY. The high rank correlations between the adjusted and non‐adjusted DC for DFI suggest that a favourable correlated impact on digestive efficiency would be conserved.

## 5 CONCLUSION

In conclusion, predicting DC from NIRS analyses of faecal samples is a promising approach to extend phenotyping of digestibility to breeding farms. Digestive coefficients of energy, organic matter and nitrogen were heritable traits with higher heritabilities estimated under a diet with increased fibre content. DC were favourably correlated with FCR, DFI and RFI from a genetic point of view. However, in future breeding schemes unfavourable genetic correlations with ADG should be carefully taken into account. Digestibility could be an interesting trait to include in breeding schemes, as pigs will be fed diets with increased fibre content in the future, to identify pigs more robust to the nature of the feed. It could also be used as a proxy of other feed efficiency traits to extend the phenotyping capacity and hence accelerate the genetic gain on these traits.

## Supporting information

Table S1‐S3

## Data Availability

The data that support the findings of this study are available on request from the corresponding author. The data are not publicly available due to privacy or ethical restrictions.
