# Supplementary material for: Genetics of digestive efficiency in growing pigs fed a conventional or a high‐fibre diet
Source: J Anim Breed Genet. 2020 Sep 20;138(2):246–58. doi: 10.1111/jbg.12506 (PMC7891433; doi:10.1111/jbg.12506)
Supplement: Supplementary file 1 — Table S1‐S3 [file JBG-138-246-s001.docx]

|  | Growing phase | |  | Finishing phase | |
| --- | --- | --- | --- | --- | --- |
| Ingredients (%) | CO diet | HF diet |  | CO diet | HF diet |
| Wheat | 42.10 | 38.00 |  | 45.10 | 39.30 |
| Corn | 25.00 | 0.00 |  | 25.00 | 0.00 |
| Barley | 10.00 | 16.90 |  | 10.00 | 17.60 |
| Rapeseed meal | 6.00 | 6.00 |  | 10.00 | 9.90 |
| Sunflower meal no shelled | 3.00 | 3.00 |  | 4.80 | 3.00 |
| Soybean meal, 48% CP | 10.40 | 5.40 |  | 2.50 | 0.00 |
| Wheat bran | 0.00 | 15.00 |  | 0.00 | 15.00 |
| Soybean hulls | 0.00 | 8.00 |  | 0.00 | 8.00 |
| Beet pulp | 0.00 | 5.00 |  | 0.00 | 5.00 |
| _L-_Lys | 0.44 | 0.35 |  | 0.11 | 0.31 |
| _DL-_Met | 0.09 | 0.03 |  | 0.01 | 0.00 |
| _L-_Thr | 0.13 | 0.11 |  | 0.02 | 0.10 |
| Pure valine | 0.02 | 0.00 |  | 0.00 | 0.00 |
| Calcium carbonate | 1.40 | 1.12 |  | 0.12 | 1.01 |
| Dicalcium phosphate | 0.49 | 0.29 |  | 0.05 | 0.00 |
| NaCl | 0.40 | 0.40 |  | 0.40 | 0.40 |
| Vitamin and trace mineral mixture 0.5% | 0.40 | 0.40 |  | 0.40 | 0.40 |

**Supplementary Table S1**: Ingredient composition (%) of the conventional (CO) and the high fibre (HF) diets

**Supplementary Table S2**: Heritability (h²), genetic and phenotypic variances of digestibility coefficients not adjusted for daily feed intake, for growing pigs fed the conventional (CO) and high fiber (HF) diet, along with their standard error (se)

|  | CO diet | | |  | HF diet | | |  | Across diets |
| --- | --- | --- | --- | --- | --- | --- | --- | --- | --- |
|  | h²  (se) | Genetic  variance  (se) | Phenotypic  variance  (se) |  | h²  (se) | Genetic variance  (se) | Phenotypic  variance  (se) |  | Genetic  correlation  (se) |
| Digestibility coefficients |  |  |  |  |  |  |  |  |  |
| Energy, % | 0.38  (0.12) | 1.99  (0.67) | 5.21  (0.43) |  | 0.54 (0.15) | 2.83  (0.87) | 5.28  (0.37) |  | 0.76  (0.15) |
| Nitrogen content, % | 0.41  (0.12) | 3.38  (1.10) | 8.31  (0.65) |  | 0.56 (0.15) | 3.29  (1.00) | 5.92  (0.42) |  | 0.79  (0.15) |
| Organic matter, % | 0.40  (0.12) | 1.75  (0.57) | 4.38  (0.37) |  | 0.54 (0.15) | 2.41  (0.75) | 4.49  (0.39) |  | 0.86  (0.16) |

**Supplementary Table S3**: Genetic correlations between digestibility coefficients (DC) not adjusted for daily feed intake and production traits for pigs fed a conventional (CO) diet or a high-fiber (HF) diet, and for the two datasets combined, along with their standard errors (se)

|  | Energy DC | |  | Nitrogen DC | |  | Organic Matter DC | |  | Diets combined | | |
| --- | --- | --- | --- | --- | --- | --- | --- | --- | --- | --- | --- | --- |
|  | CO diet | HF diet |  | CO diet | HF diet |  | CO diet | HF diet |  | Energy | Nitrogen | Organic Matter |
| Growth traits |  |  |  |  |  |  |  |  |  |  |  |  |
| FCR, kg/kg | -0.59 (0.23) | -0.20 (0.37) |  | -0.36 (0.23) | -0.37 (0.23) |  | -0.56 (0.23) | -0.25 (0.36) |  | -0.39 (0.14) | -0.39 (0.09) | -0.43 (0.13) |
| DFI, kg/day | -0.78 (0.16) | -0.60 (0.26) |  | -0.69 (0.16) | -0.48 (0.26) |  | -0.78 (0.16) | -0.61 (0.26) |  | -0.75 (0.10) | -0.64 (0.10) | -0.66 (0.10) |
| ADG, g/day | -0.52 (0.21) | -0.65 (0.30) |  | -0.62 (0.20) | -0.42 (0.29) |  | -0.53 (0.20) | -0.60 (0.30) |  | -0.53 (0.13) | -0.45 (0.13) | -0.42 (0.13) |
| RFI, g/day | -0.99^1^ | -0.62 (0.23) |  | -0.86 (0.30) | -0.57 (0.22) |  | -0.99^1^ | -0.63 (0.23) |  | -0.65 (0.12) | -0.64 (0.12) | -0.65 (0.12) |
| Carcass Composition |  |  |  |  |  |  |  |  |  |  |  |  |
| LMP, % | 0.31 (0.23) | -0.11 (0.22) |  | 0.25 (0.22) | -0.06 (0.23) |  | 0.34 (0.22) | -0.05 (0.24) |  | 0.19 (0.15) | 0.16 (0.14) | 0.18 (0.14) |
| Carcass Yield, % | -0.28 (0.25) | -0.03 (0.24) |  | -0.21 (0.25) | -0.20 (0.21) |  | -0.24 (0.25) | -0.15 (0.22) |  | -0.19 (0.17) | -0.31 (0.21) | -0.19 (0.17) |
| BellyP, % | 0.05 (0.31) | 0.21 (0.33) |  | 0.03 (0.29) | 0.07 (0.34) |  | -0.02 (0.30) | 0.16 (0.34) |  | 0.13 (0.18) | 0.08 (0.17) | 0.12 (0.17) |
| LoinP, % | 0.08 (0.35) | -0.27 (0.33) |  | -0.06 (0.35) | -0.10 (0.34) |  | 0.09 (0.34) | -0.16 (0.34) |  | 0.03 (0.21) | 0.01 (0.20) | 0.03 (0.21) |
| ShoulderP, % | -0.33 (0.27) | -0.10 (0.25) |  | -0.16 (0.27) | -0.05 (0.25) |  | -0.31 (0.27) | -0.10 (0.26) |  | -0.17 (0.16) | -0.14 (0.15) | -0.16 (0.16) |
| BackfatP, % | -0.28 (0.24) | -0.08 (0.23) |  | -0.26 (0.23) | -0.05 (0.23) |  | -0.31 (0.23) | 0.00 (0.11) |  | -0.20 (0.14) | -0.17 (0.14) | -0.19 (0.14) |
| HamP, % | 0.46 (0.25) | 0.22 (0.28) |  | 0.39 (0.23) | 0.10 (0.12) |  | 0.53 (0.24) | 0.18 (0.29) |  | 0.19 (0.16) | 0.13 (0.16) | 0.19 (0.16) |
| Meat Quality |  |  |  |  |  |  |  |  |  |  |  |  |
| upH | -0.83 (0.32) | -0.36 (0.38) |  | -0.54 (0.33) | -0.61 (0.39) |  | -0.81 (0.32) | -0.38 (0.38) |  | -0.40 (0.23) | -0.21 (0.21) | -0.38 (0.22) |
| L* | -0.02 (0.32) | -0.14 (0.39) |  | -0.22 (0.30) | 0.35 (0.47) |  | 0.00 (0.32) | -0.10 (0.40) |  | -0.15 (0.21) | -0.07 (0.22) | -0.16 (0.21) |
| a* | 0.12 (0.09) | -0.16 (0.22) |  | 0.15 (0.22) | -0.20 (0.21) |  | 0.27 (0.22) | -0.21 (0.21) |  | 0.10 (0.07) | -0.14 (0.14) | -0.14 (0.14) |
| b* | 0.21 (0.34) | -0.19 (0.32) |  | 0.01 (0.32) | 0.01 (0.33) |  | 0.19 (0.33) | -0.22 (0.32) |  | -0.16 (0.18) | -0.14 (0.18) | -0.19 (0.17) |

FCR = Feed Conversion Ratio; DFI = Daily Feed Intake; ADG = Average Daily Gain; RFI = Residual Feed Intake; LMP = Lean Meat Percentage BellyP = Belly Percentage; LoinP = Loin Percentage; ShoulderP = Shoulder Percentage; BackfatP = Backfat Percentage; HamP = Ham Percentage; upH = ultimate pH 24 hours after the slaughterhouse; L = lightness of the meat; a* = redness of the meat; b* = yellowness of the meat

^1^ Estimated correlation and at the edge of the parameter space
